# A Prospective, Open-label, Randomized Trial of Doxycycline Versus Azithromycin for the Treatment of Uncomplicated Murine Typhus

**DOI:** 10.1093/cid/ciy563

**Published:** 2018-07-18

**Authors:** Paul N Newton, Valy Keolouangkhot, Sue J Lee, Khamla Choumlivong, Siho Sisouphone, Khamloune Choumlivong, Manivanh Vongsouvath, Mayfong Mayxay, Vilada Chansamouth, Viengmon Davong, Koukeo Phommasone, Joy Sirisouk, Stuart D Blacksell, Pruksa Nawtaisong, Catrin E Moore, Josée Castonguay-Vanier, Sabine Dittrich, Sayaphet Rattanavong, Ko Chang, Chirapha Darasavath, Oudayvone Rattanavong, Daniel H Paris, Rattanaphone Phetsouvanh

**Affiliations:** 1 Lao-Oxford-Mahosot Hospital–Wellcome Trust Research Unit, Microbiology Laboratory, Mahosot Hospital, Vientiane, Lao People’s Democratic Republic; 2 Centre for Tropical Medicine & Global Health, University of Oxford, United Kingdom; 3 Adult Infectious Disease Ward, Mahosot Hospital, Vientiane, Lao People’s Democratic Republic; 4 Mahidol Oxford Research Unit, Faculty of Tropical Medicine, Mahidol University, Bangkok, Thailand; 5 Setthathirat Hospital, Vientiane, Lao People’s Democratic Republic; 6 Faculty of Postgraduate Studies, University of Health Sciences, Vientiane, Lao People’s Democratic Republic; 7 Department of Medicine, Swiss Tropical and Public Health Institute, Switzerland; 8 Faculty of Medicine, University of Basel, Switzerland

**Keywords:** murine typhus, *Rickettsia typhi*, Laos, doxycycline, azithromycin

## Abstract

**Background:**

Murine typhus, or infection with *Rickettsia typhi*, is a global but neglected disease without randomized clinical trials to guide antibiotic therapy.

**Methods:**

A prospective, open, randomized trial was conducted in nonpregnant, consenting inpatient adults with rapid diagnostic test evidence of uncomplicated murine typhus at 2 hospitals in Vientiane, Laos. Patients were randomized to 7 days (D7) or 3 days (D3) of oral doxycycline or 3 days of oral azithromycin (A3). Primary outcome measures were fever clearance time and frequencies of treatment failure and relapse.

**Results:**

Between 2004 and 2009, the study enrolled 216 patients (72 per arm); 158 (73.2%) had serology/polymerase chain reaction (PCR)–confirmed murine typhus, and 52 (24.1%) were *R. typhi* PCR positive. The risk of treatment failure was greater for regimen A3 (22.5%; 16 of 71 patients) than for D3 (4.2%; 3 of 71) or D7 (1.4%; 1 of 71) (*P* < .001). Among *R. typhi* PCR–positive patients, the area under the time-temperature curve and the fever clearance time were significantly higher for A3 than for D3 (1.8- and 1.9-fold higher, respectively; *P* = .005) and D7 (1.5- and 1.6-fold higher; *P* = .02). No patients returned with PCR-confirmed *R. typhi* relapse.

**Conclusion:**

In Lao adults, azithromycin is inferior to doxycycline as oral therapy for uncomplicated murine typhus. For doxycycline, 3- and 7-day regimens have similar efficacy. Azithromycin use in murine typhus should be reconsidered. Investigation of genomic and phenotypic markers of *R. typhi* azithromycin resistance is needed.

**Clinical Trial Registration:**

ISRCTN47812566.

## Teaser

A clinical trial of murine typhus, or infection with *Rickettsia typhi*, in Lao adults suggests that azithromycin is inferior to doxycycline for the oral therapy of uncomplicated murine typhus. Three and 7 days of doxycycline have similar efficacy.

Murine typhus, caused by *Rickettsia typhi*, is a neglected global flea-borne disease with sparse worldwide data on epidemiology and no randomized clinical trials to guide therapy [1, 2]. Although most commonly a febrile illness with few localizing signs, it also causes severe disease, such as meningoencephalitis and pneumonitis [3]. The diagnosis of *R. typhi* infection is difficult, resembling many other causes of fever, but recent reports highlight its global importance [1–6]. *R. typhi* is in the same antigenic group as *Rickettsia prowazekii*, the cause of epidemic typhus and a more severe disease [6].

Tetracyclines are the mainstays of treatment, but there is minimal evidence on optimal duration or how to treat in pregnancy, childhood, or severe disease [1, 2, 4, 6, 7]. A 5–10-day chloramphenicol course or a single 200-mg oral doxycycline dose resulted in defervescence within 2 days in about 70% of Thai patients with murine typhus [8]. A review of Cretan patients with murine typhus suggested that doxycycline was associated with a shorter fever clearance time (FCT) than chloramphenicol or fluoroquinolones [9]. The clinical efficacy of fluoroquinolones is in doubt [10–12], with equivocal evidence for azithromycin [13, 14].

*R. typhi* antibiotic susceptibility testing cannot be assessed by conventional techniques. The few genotypic and phenotypic susceptibility data available suggest that *R. typhi* is susceptible to tetracyclines, chloramphenicol, azithromycin, erythromycin, clarithromycin, and fluoroquinolones and resistant to amoxicillin, cotrimoxazole, and gentamicin [6, 7, 15–17]. Resistance to rifampin has been described, associated with *rpoB* gene point mutations [18]. Azithromycin has been shown to be efficacious by means of in vitro assays against diverse *Rickettsia* species [19]. We therefore conducted a randomized open-label clinical trial to provide evidence for the optimum duration of treatment of murine typhus in Laos and to test azithromycin as an alternative therapy.

## PATIENTS AND METHODS

### Study Design

An open, randomized, superiority trial was performed in adults with rapid diagnostic test (RDT) evidence of infection with uncomplicated murine typhus, to compare the therapeutic efficacy of 3 oral treatment regimens: 7 days of doxycycline (D7), 3 days of doxycycline (D3), and 3 days of azithromycin (A3). The study was conducted at 2 primary-tertiary care hospitals in Vientiane: Mahosot Hospital (400 beds) and Sethathirat Hospital (200 beds). Ethical clearance was granted by the Ethical Review Committee of the Faculty of Medical Sciences, National University of Laos, Vientiane, and the Oxford Tropical Research Ethics Committee in the United Kingdom.

Eligible participants admitted to the infectious disease wards at both hospitals were screened and enrolled by study physicians. Nonpregnant adults (aged ≥15 years) admitted with suspected uncomplicated typhus were included in the study providing they had a positive anti–*R. typhi* immunoglobulin M (IgM) result, were able to take oral medication, gave written informed consent, and had a high likelihood of remaining in the hospital for the duration of treatment and completing ≥4 weeks of follow-up. A negative urinary pregnancy test was required for women of childbearing age. 

Exclusion criteria included known administration of chloramphenicol, doxycycline, tetracycline, fluoroquinolones, or azithromycin during the week before admission; pregnancy or breastfeeding; and hypersensitivity or contraindications to doxycycline (severe hepatic impairment, known systemic lupus erythematosus) or azithromycin (severe hepatic impairment). Patients were also excluded if they had evidence of another cause of fever or severe disease, defined as ≥1 of the following: reduced level of consciousness, jaundice, systolic blood pressure <80 mm Hg, vomiting, respiratory distress (respirations, >30/min), or any other syndrome that in the opinion of the admitting physician constituted severe typhus. Patients were not involved in the design of this trial. (Clinical trial registration: ISRCTN47812566.)

### Randomization

Patients were randomized to 3 groups: (1) D7, doxycycline with a 200-mg loading dose, followed by 100 mg every 12 hours for 7 days; (2) D3, doxycycline with a 200-mg loading dose, followed by 100 mg every 12 hours for 3 days; or (3) A3, azithromycin with a 500-mg loading dose, followed by 250 mg every 24 hours for 2 days.

The drugs used were doxycycline hyclate (Vibramycin, 100-mg film-coated tablets; Pfizer) and azithromycin dihydrate (Zithromax, 250-mg capsules; Pfizer). Randomization, in blocks of 12, was computer generated by an investigator not involved in patient recruitment. Treatment allocations were kept in sealed opaque envelopes and opened, by recruiting study physicians, when all inclusion and exclusion criteria were checked and consent forms signed. Paracetamol was given for fever as needed by the patient and antacids avoided. The ingestion of all study drugs was witnessed by the ward nursing staff. If a patient vomited within 1 hour after drug ingestion, the full dose was repeated.

### Procedures

All consenting patients with a history of fever and suspected typhus had venous blood taken and aliquoted for serum (3 mL) and whole blood in ethylenediaminetetraacetic acid (EDTA) (5 mL) for a full blood count (Abx Micros 60; Abx Hematologie) and 2 blood cultures (5 mL in a 50-mL blood culture bottle) that were incubated in air at 37°C for 7 days (Table 1). Bacteria isolated from blood cultures were identified using standard biochemical tests and specific antisera. If the patient came from an area with endemic malaria, Giemsa-stained malaria thick and thin films were examined. 

**Table 1.** Clinical and Laboratory Features of Patients Recruited to Murine Typhus Clinical Trial

Variable^a^	All Patients	*Rickettsia typhi* Positive	
		Serology and/or PCR	PCR
Patients, No. (%)	216 (100)	158 (73.2)^b^	52 (24.2)^c^
Age, median (IQR), y	30 (22–41)	30 (21–42)	39 (22–47)
Female sex, No. (%)	91 (42.1)	61 (38.6)	20 (38.5)
Duration of illness, median (IQR), d	8 (7–10)	8 (7–10)	8 (7–10)
Symptoms, No. (%)			
Headache	204/215^d^ (94.9)	152/157 (96.8)	51 (98.1)
Myalgia	164 (75.9)	122 (77.2)	45 (86.5)
Abdominal pain	15/215 (7.0)	12/157 (7.6)	6 (11.5)
Nausea	63 (29.2)	48 (30.4)	16 (30.8)
Vomiting	60 (27.8)	45 (28.5)	16 (30.8)
Diarrhea	58 (26.9)	44 (27.9)	21 (40.4)
Cough	97 (44.9)	69 (43.7)	24 (46.2)
Lymphadenopathy	28/215 (13.0)	21/157 (13.4)	9 (17.3)
Rash	41 (19.0)	31 (19.6)	14 (26.9)
Liver palpable	19 (8.8)	12 (7.6)	4 (7.7)
Spleen palpable	13 (6.0)	10 (6.3)	4 (7.7)
Tympanic temperature, mean (95% CI), °C	38.5 (38.4–38.7)	38.6 (38.4–38.7)	38.7 (38.5–38.9)
Pulse rate, median (IQR), pulses/min	98 (88–100) (n = 215)^d^	98 (88–100) (n = 157)	100 (89–100)
Systolic BP, median (IQR), mm Hg	100 (100–120)	100 (100–120)	100 (95–120)
Diastolic BP, median (IQR), mm Hg	70 (60–80)	70 (60–80)	70 (60–80)
Respiratory rate, mean (95% CI), respirations/min	22.3 (21.5–23.0) (n = 213)	22.3 (21.3–23.4) (n = 155)	22.4 (21.4–23.4)
Body weight, mean (95% CI), kg	54.6 (53.2–55.9)	55.7 (54.1–57.2)	57.4 (54.5–60.3)
Hematocrit, mean (95% CI), %	40.6 (39.9–41.4) (n = 215)	40.7 (39.8–41.7) (n = 157)	40.1 (38.6–41.6) (n = 51)
WBC count, median (IQR), 10^9^/L	8.3 (6.4–10.3) (n = 214)	7.9 (6.4–9.9) (n = 156)	7.8 (5.8–9.9) (n = 51)
Neutrophils, mean (95% CI), %	64.8 (63.2–66.4) (n = 215)	64.3 (62.7–65.9) (n = 157)	66.0 (63.0–69.0) (n = 51)
Platelet count, mean (95% CI), 10^9^/L	192 (185–199) (n = 213)	191 (183–200) (n = 155)	187 (169–205) (n = 51)
Serum CRP, median (IQR), mg/L	37.6 (18.5–83.3) (n = 197)	34.9 (18.5–76.0) (n = 147)	66.9 (21.9–111.9) (n = 49)
Serum creatinine, median (IQR), μmol/L	97 (80–106) (n = 205)	97 (80–106) (n = 153)	97 (80–115)
Serum AST, median (IQR), IU/L	91 (50–165) (n = 200)	101 (58–167) (n = 149)	141 (84–187) (n = 49)
Serum ALT, median (IQR), IU/L	33 (18–61) (n = 197)	36 (22–69) (n = 147)	42 (27–71) (n = 49)
Serum albumin, median (IQR), g/L	3.6 (3.2–4.0) (n = 202)	3.6 (3.2–4.0) (n = 151)	3.2 (2.8–3.6) (n = 51)
Serum alkaline phosphatase, median (IQR), IU/L	105 (74–168) (n = 199)	119 (77–182) (n = 149)	168 (96–260) (n = 49)
Serum total bilirubin, median (IQR), μmol/L	5.1 (5.1–8.6) (n = 198)	5.1 (5.1–8.6) (n = 147)	6.8 (5.1–13.7) (n = 49)

Abbreviations: ALT, alanine aminotransferase; AST, aspartate aminotransferase; BP, blood pressure; CRP, C-reactive protein; IQR, interquartile range; PCR, polymerase chain reaction; WBC, white blood cell.

^a^Reference ranges for laboratory values were as follows: WBC count, 4.0–11.0 10^9^/L; platelet count, 150–400 10^9^/L; serum CRP, <10 mg/L; serum creatinine, 53–123 μmol/L; serum AST, 7–40 IU/L; serum ALT, 7–40 IU/L; serum albumin, 3.5–5.0 g/L; serum alkaline phosphatase 24–190 IU/L; and serum total bilirubin, 1.7–20 μmol/L.

^b^A total of 163 patients had serologic or PCR evidence of *R. typhi* infection, but 5 had PCR/culture evidence of another pathogen (*Escherichia coli*, *Salmonella* Typhi, *Leptospira* spp. in 1 each and *Orientia tsutsugamushi* in 2). They may have had concurrent dual infections, but we have opted to be conservative and not included these 5 patients, giving a denominator of 158 patients

^c^A total of 53 patients had PCR evidence of *R. typhi* infection, but 1 patient was also PCR positive for *Leptospira* spp., with an apparent dual concurrent infection, and is thus excluded from this column.

^d^Denominators are provided for variables with missing values.

Samples were stored at −80°C until analysis and transported on dry ice. IgM antibodies to murine typhus were screened by the Dip-S-Ticks Murine typhus (D-RTY03T, PanBio, now GenBio, adapted by detecting IgM). These have a sensitivity and specificity for diagnosing murine typhus of 61% and 87%, respectively, compared with indirect immunofluorescence assays (IFAs) [20]. IgM and immunoglobulin G (IgG) antibodies against *R. typhi* were detected using IFA after the study was completed, using slides coated with *R. typhi* Wilmington antigen (Australian Rickettsial Reference Laboratory). Patient serum samples were serially 2-fold diluted from 1:400 to 1:3200, and the end point was determined as the highest titer displaying specific fluorescence. Positivity was defined as (1) ≥4-fold rising titer in IgM or IgG antibodies when comparing admission with subsequent longitudinal samples and/or (2) a positive reciprocal titer of ≥3200 in an admission sample.

EDTA buffy coat samples underwent genomic DNA extraction using the QIAmp DNA Mini Kit (Qiagen), followed by detection of the 47-kDa *htrA* gene of *Orientia tsutsugamushi*, 17-kDa gene of *Rickettsia* spp., and the *rrs* gene of *Leptospira* spp. (see Supplementary Material for details).

Patients’ admission clinical history and examination were recorded using standard questionnaires and reviewed daily. Tympanic temperature was recorded every 6 hours, using tympanic thermometers (Genius; Tyco Healthcare). For those Mahosot Hospital patients who consented, follow-up was conducted on days 14 and 28 and at 3, 6, and 12 months, with repeated venous blood collection. Patients were encouraged to return if they felt unwell within the year after discharge.

### Outcomes

The primary outcome measures were FCT, and frequencies of treatment failure and confirmed relapse with, as secondary outcome, area under the time-temperature curve (AUC). FCT was defined as the time, from onset of treatment, to the first time tympanic temperature dropped below 37.5°C, after a rise to ≥37.5°C, and remained at ≤37.5°C for 24 hours. FCT spanned primary and rescue therapy for those with treatment failure, which was defined as fever >37.5°C after ≥72 hours of the assigned treatment without clinical improvement or with development of severe disease. Patients in whom first-line treatments failed were treated with 7 days doxycycline (100 mg every 12 hours with loading dose, as described above). Detailed clinical history, examination, and investigations were performed to determine the cause of fever in those with apparent “relapse.” Confirmed relapse was defined as a patient presenting again with fever and *R. typhi* buffy coat polymerase chain reaction (PCR) positivity because it was not possible to reliably clinically distinguish *R. typhi* relapse from other causes of fever, and serology responses in *R. typhi* relapse are not known.

### Statistical Analysis

Without local data on murine typhus FCT and treatment failure, sample size calculations were performed at interim analysis after 51 patients; there were no failures in the D7 group (0 of 18 patients), a 12.5% failure rate (2 of 16 patients) in the D3 group, and a 29.4% failure rate (5 of 17) in the A3 group. To detect a significant difference in the proportion of failures between the reference group (using 0.1%) and the D3 group, a sample size of 67 per group provided 80% power and an α value of .05, with sufficient power to detect a difference between the D7 and A3 groups as well. To account for potential patient withdrawal, 72 patients per group were recruited (n = 216 total).

Outcomes were compared across the 3 treatment groups and also by subgroups: serology- or PCR-confirmed *R. typhi* (single infection) and PCR-confirmed *R. typhi*. We regarded PCR positivity as a method with higher specificity to diagnose *R. typhi* than serology. Frequencies were reported as numbers and percentages and analyzed using χ^2^ test. Risks were quantified using logistic regression and treatment allocation as a categorical covariate, with D7 serving as the referent group. Median FCTs were estimated using survival analysis and plotted using Kaplan-Meier curves. The survival functions were compared using the log-rank test. AUC was calculated using the trapezoid rule to the time when temperature dropped below 37.5°C and stayed at ≤37.5°C for ≥24 hours. For patients whose fever did not clear, the time of discharge was used. Patients who were afebrile (and stayed afebrile) at admission were excluded from the AUC analysis. AUCs were compared between the 3 treatment groups using the Kruskal-Wallis test. Analysis was performed using Stata software (version 14.2; StataCorp).

## RESULTS

**Figure 1.**
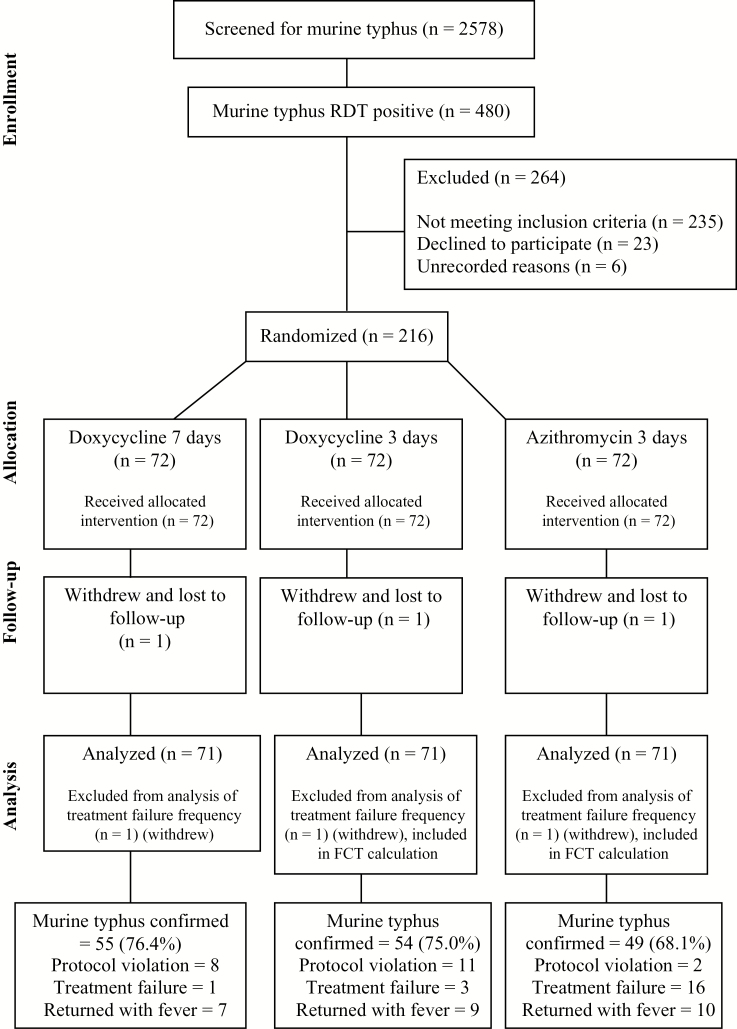
Flowchart of the clinical trial. Patients were excluded (n = 264) because they were unlikely to be able to stay on the ward for 7 days and/or complete follow-up (95 patients; 36.0%), had severe disease (50; 18.9%), had taken antirickettsial antibiotics (31; 11.7%), were not admitted to the study wards (27; 10.2%), declined consent (23; 8.7%), were pregnant or breastfeeding (11; 4.2%), were children (10; 3.8%), or had an alternative confirmed diagnosis before being approached for consent (8; 3.0%); 6 patients (2.3%) were excluded for unrecorded reasons, and 3 (1.1%) withdrew from the study. The confirmed totals included those without culture or polymerase chain reaction evidence of dual pathogens.

Between 21 March 2004 and 13 August 2009, a total of 2313 and 265 inpatients at Mahosot and Sethathirat Hospitals, respectively, were screened using anti–*R. typhi* IgM RDTs; 480 had murine typhus diagnosed, 416 (87%) at Mahosot Hospital and 64 (13%) at Sethathirat (Figure 1). Two hundred sixteen patients (45%; 72 patients in each treatment group) were recruited into the trial (180 at Mahosot, 36 at Sethathirat Hospital); 91 (42%) were female, with a median age of 30 years (interquartile range [IQR], 22–41 years) (Table 1). The main reasons for exclusion (n = 264) were that patients were unlikely to be able to stay on the ward for 7 days and/or complete follow-up (95 patients; 36.0%), had severe disease (50; 18.9%), had taken antirickettsial antibiotics (31; 11.7%), were not admitted to the study wards (27; 10.2%), declined consent (23; 8.7%), or were pregnant or breastfeeding (11; 4.2%) (Figure 1). Three patients, 1 in each treatment group, withdrew.

**Figure 2.**
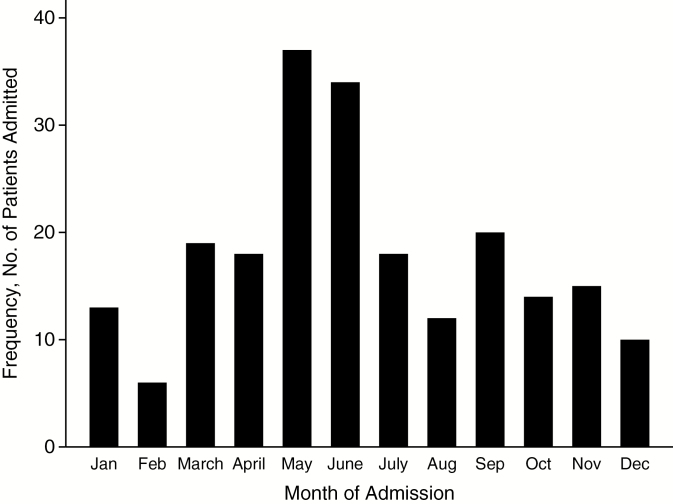
Monthly recruitment of patients by admission month. Abbreviations: Aug, August; Dec, December; Feb, February; Jan, January; Nov, November; Oct, October; Sep, September.

Patients were recruited in all months, with peaks in May–June (Figure 2). The trial ended when the target of 216 patients was enrolled. Most patients lived in Vientiane City (199 patients; 92%) and Vientiane Province (15; 6.9%). The most common occupations were student (50 patients; 23.1%), trader (38; 17.6%), construction worker (20; 9.3%), and government official (19; 8.8%). Of 204 patients with data, 91 (45%) stated that they had taken antibiotics in the week before admission; none had taken an antibiotic known to be active against *R. typhi*.

Of the 216 patients, 158 (73.2%) had murine typhus confirmed by IFA and/or PCR, without evidence of dual infections, including 52 of 216 (24.1%) with PCR lone-infection evidence of *R. typhi*. The median (IQR) bacterial load in 45 *R. typhi* PCR–positive patients was 3 (2–6) copies/μL whole blood (Tables 2 and 3).

Of 160 patients (74.1%) with IgM/IgG serologic evidence of *R. typhi* infection, 5 had PCR/culture evidence of another pathogen (*Escherichia coli*, *Salmonella enterica* serovar Typhi, and *Leptospira* sp. in 1 each and *O. tsutsugamushi* in 2) (Tables 2 and 3). These patients may have had concurrent genuine dual infections and were not included in the analysis.

Pathogens were identified by PCR for 62 patients (28.7%): 53 with *R. typhi,* 6 with *O. tsutsugamushi,* and 4 with *Leptospira* spp. One patient was PCR positive for both *R. typhi* and *Leptospira* sp. Another 5 patients without IFA IgM and IgG evidence of *R. typhi* were blood culture positive (4 with *Salmonella* Typhi and 1 with *Salmonella* Paratyphi A) (Table 2). At examination, 3 patients (1.3%) had eschars; all were PCR positive for *O. tsutsugamushi*. Malaria films obtained in 183 patients (85%) were negative. Three patients with positive anti–*R. typhi* IgM RDT and *R. typhi* PCR results had negative *R. typhi* IgM and IgG IFA results (see Patients and Methods).

**Table 2.** Pathogen Identification for the 175 Patients of 216 With Laboratory Diagnoses, Recruited to a Murine Typhus Clinical Trial

Pathogen	Patients, No.			
	Total	Culture	PCR	Serology
*Rickettsia typhi*	158^a,b^	NA	53^a^	155^b^
*Orientia tsutsugamushi*	7	NA	6	4
*Salmonella enterica* Typhi^c^	5	5	NA	NA
*Leptospira* spp.	4	NA	4^a^	NA
*Escherichia coli* ^c^	1	1	NA	NA
*Salmonella enterica* Paratyphi A^c^	1	1	NA	NA

Abbreviations: NA, not applicable; PCR, polymerase chain reaction.

^a^One patient was PCR positive for both *R. typhi* and *Leptospira* sp.

^b^A total of 160 patients had serologic evidence of *R. typhi* infection, but 5 had PCR/culture evidence of another pathogen (*E. coli*, *Salmonella* Typhi, and *Leptospira* sp. in 1 each and *O. tsutsugamushi* in 2). They may have had concurrent dual infections, but we have opted to be conservative and not included these 5 patients, giving a denominator of 155 patients.

^c^Blood culture and bacterial identification as described by Phetsouvanh et al [21].

Seventy-two patients were randomly assigned to each of the 3 treatment groups (Table 3 and Figure 1). Of the 158 patients with confirmed single-pathogen murine typhus infection, 55 (34.8%), 54 (34.2%), and 49 (31.0%) were included in the D7, D3, and A3 groups, respectively.

**Table 3.** Details of Patients Recruited to the 3 Treatment Groups^a^

Variable	Doxycycline (7 d) (n = 72)	Doxycycline (3 d) (n = 72)	Azithromycin (3 d) (n = 72)	All Patients (n = 216)
Clinical and laboratory features				
Age, median (IQR), y	28.5 (21–43)	29.0 (21–40)	31.5 (24–42)	30 (22–41)
Female sex, No. (%)	33 (45.8)	30 (41.7)	28 (38.9)	91 (42.1)
Duration of illness, median (IQR), d	8 (7–10)	9 (7–10)	8 (7–10)	8 (7–10)
Fever ≥37.5°C at admission, No. (%)	61 (84.7)	64 (88.9)	68 (94.4)	193 (89.4)
Admission tympanic temperature, mean (95% CI), °C	38.6 (38.3–38.8)	38.4 (38.2–38.6)	38.6 (38.4–38.8)	38.5 (38.4–38.7)
PCR- or serology-confirmed *Rickettsia typhi* , No. (%)^b^	55 (76.4)	54 (75.0)	49 (68.1)	158 (73.2)
Serology-confirmed *R. typhi*, No. (%)	53/70^c^ (75.7)	56/72 (77.8)	51/70 (72.9)	160/212 (75.5)
PCR-confirmed *R. typhi*, No. (%)^b^	20/71 (28.2)	18/69 (26.1)	15/71 (21.1)	53/211 (25.1)
No PCR or serologic evidence of *R. typhi*, No. (%)	17 (23.6)	18 (25.0)	23 (31.9)	58 (26.9)
*R. typhi* bacteremia, median (IQR), copies/μL whole blood	3 (2–6) (n = 17)c	3 (2–4) (n = 15)	3 (2–4) (n = 13)	3 (2–6) (n = 45)
PCR- or serology-confirmed *O. tsutsugamushi*, No. (%)	0/69	5/69 (7.3)	2/69 (2.9)	7/207 (3.4)
PCR-confirmed *O. tsutsugamushi*, No. (%)	0/71	4/69 (5.8)	2/71 (2.8)	6/211 (2.8)
Other confirmed diagnoses, No. (%)	6/72 (8.3)	2/72 (2.8)	3/72 (4.2)	11/216 (5.1)
No diagnosis made, No. (%)	12/72 (16.7)	11/72 (15.3)	18/72 (25.0)	41/216 (19.0)
CRP, median (IQR), mg/L	40.8 (22.8–94.5) (n = 66)^c^	34.2 (14.0–95.2) (n = 63)	35.7 (19.0–59.4) (n = 68)	37.6 (18.5–83.3) (n = 197)
Antibiotic dosages				
Total doxycycline doses administered, median (range), No.^d^	15 (2–17)	7 (4–21)	0 (0–15)	…
Total duration of doxycycline treatment, median (range), d^d^	7 (1–8)	3.5 (2–10)	0 (0–7)	…
Doxycycline dosage, mean (95% CI), mg/kg body weight dose per 100-mg dose	1.83 (1.75–1.92)	1.83 (1.76- 1.91)	1.84 (1.77–1.92) (n = 14)c	_…_
Total doxycycline dose, mean (95% CI), mg/kg body weight^d^	27.5 (26.0–29.0)	14.0 (13.1–15.0)	4.5 (2.3–6.7)	…
Total azithromycin doses administered, median (range), No.	0	0	4 (4–4)	…
Total duration of azithromycin treatment, median (range), d	0	0	3 (3–3)	…
Azithromycin dosage, mean (95% CI), mg/kg body weight dose per 250-mg dose	0	0	4.60 (4.42–4.79)	…
Total azithromycin dose, mean (95% CI), mg/kg body weight	…	…	18.9 (18.1–19.6)	…
Outcome				
Patients withdrawing, No. (%)	1/72 (1.4)	1/72 (1.4)	1/72 (1.4)	3/216 (1.4)
Patients vomiting loading dose within 1 h, No. (%)	5/66 (7.6)	5/64 (7.8)	2/62 (3.2)	12/192 (6.3)
Patients with mild adverse events, excluding headache, No. (%)^e^	26/36 (72)	17/37 (46)	16/30 (53)	59/103 (57)
Duration of patient follow-up, median (range), d	190 (2–666)	319 (4–411)	224 (2–420)	208 (2–666)
Patients returning with fever after admission, No. (%)	7 (9.7)	9 (12.5)	10 (13.9)	26 (12.0)

Abbreviations: CI, confidence interval; CRP, C-reactive protein; IQR, interquartile range; PCR, polymerase chain reaction.

^a^Antibiotic batch numbers were as follows: Vibramycin (Pfizer), 0658105B, 0558103B, 0758103C, 0558103D, 0558101C, and 0458102B; Zithromax (Pfizer), 914640271, 814360231, 614646251, 616046261, 516645351, 714641322, 514645351, 214642178, 514665081, 014640091, and 516645351.

^b^Without culture or PCR evidence of dual pathogens.

^c^Denominators are provided for variables with missing values.

^d^Including doxycycline given to those with treatment failure.

^e^Mahosot Hospital patients only.

The mean total doxycycline doses were 27.5 (95% CI, 26.0–29.0) and 14.0 (13.1–15.0) mg/kg body weight for D7 and D3 patients, respectively. For the A3 group, the mean total azithromycin dose was 18.9 (95% CI, 18.1–19.6) mg/kg (Table 3). There were 17 protocol violations and 3 patients withdrew, not allowing classification for treatment failure (Figure 1 and Supplementary Material). Twelve of 192 patients (6.3%) with data vomited within 1 hour of the loading dose, 3.2% (2 of 62) in the A3 group and 7.7% (10 of 130) in the D7 and D3 groups combined (*P* = .34).

**Figure 3.**
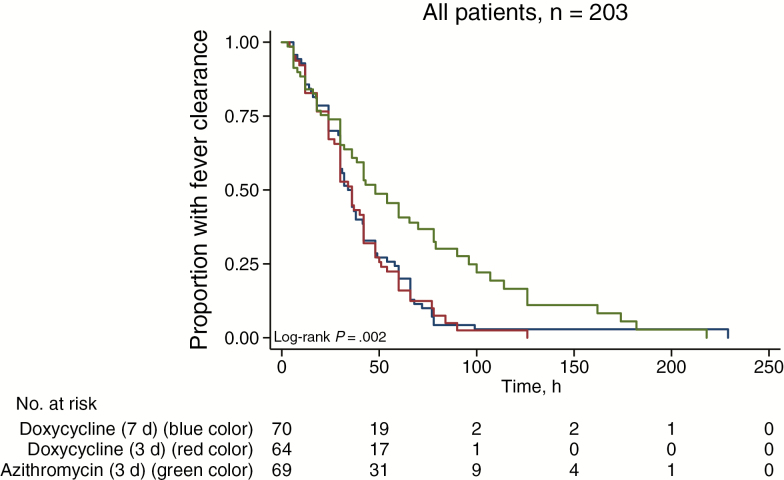
Kaplan-Meier plot of fever clearance for all patients who presented with or developed fever (n = 203).

**Figure 4.**
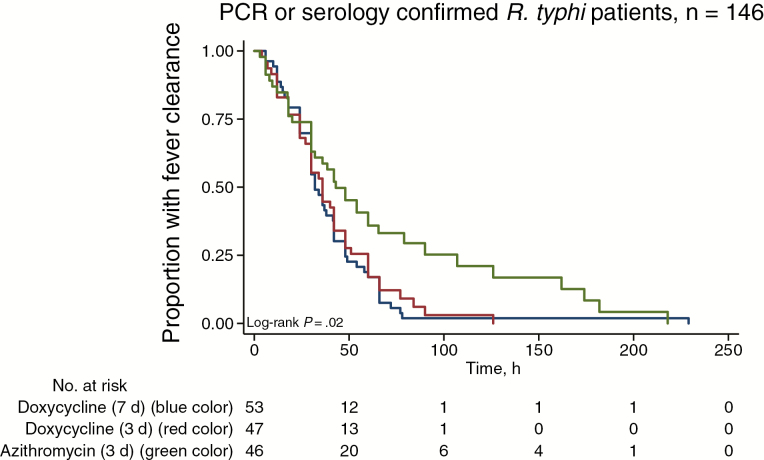
Kaplan-Meier plot of fever clearance for patients with serology- or polymerase chain reaction (PCR)–confirmed murine typhus (n = 146).

**Figure 5.**
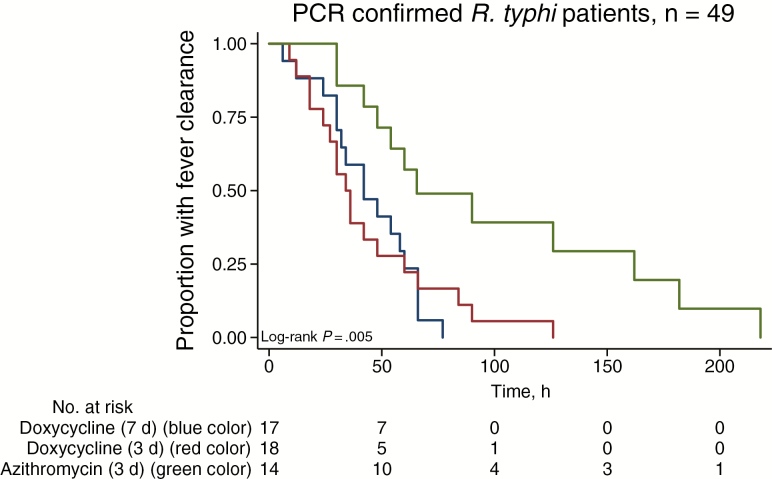
Kaplan-Meier plot of fever clearance for patients with polymerase chain reaction (PCR)–confirmed murine typhus only (n = 49).

All patients survived to discharge. The median duration of hospital admission was 8 days (IQR, 7–10 days; range, 2–90 days). Treatment failed in 20 (9.4%) of 213 patients (3 withdrew); 1 of 71 (1.4%) in the D7 group, 3 of 71 (4.2%) in the D3 group, and 16 of 71 (22.5%) in the A3 group (*P* < .001; Table 4). The risk of treatment failure (Table 4 and Supplementary Material) was also significantly higher in the A3 group than in the D7 and D3 groups when only those with molecular and/or serologically confirmed *R. typhi* (*P* = .001; n = 157) and those PCR positive for *R. typhi* (*P* = .002; n = 51) were considered. Those who failed treatment did not have only raised temperature but were also clinically unwell; none developed severe disease (Supplementary Material). The number of patients who cleared fever by 72 hours was also significantly lower in the A3 group for all patients (*P* = .001) and those *R. typhi* PCR and/or serology positive (*P* = .001). The median (IQR) FCT was significantly longer in the A3 group, at 48 (IQR, 24–96) hours, compared with 34 (24–58) hours for the D7 and 36 (24–51) hours for the D3 group (*P* = .002) (Table 4 and Figures 3–5).

**Table 4.** Outcome Measures in the Patients Recruited to a Murine Typhus Clinical Trial

Outcome Measure	All	Doxycycline (7 d)	Doxycycline (3 d)	Azithromycin (3 d)	P Value^a^
Treatment failure, No. (%)^b^					
All patients	20/213 (9.4)	1/71 (1.4)	3/71 (4.2)	16/71 (22.5)	<.001
PCR- or serology-confirmed *Rickettsia typhi*	11/157 (7.0)	0/54	2/54 (3.7)	9/49 (18.4)	<.001
PCR-confirmed *R. typhi*	7/51 (13.7)	0/18	1/18 (5.6)	6/15 (40.0)	.002
No PCR or confirmed serologic evidence of R. typhi	9/56 (16.1)	1/17 (5.88)	1/17 (5.88)	7/22 (31.8)	.06
Cleared fever, No. (%)^c^					
All patients	185/203 (91.1)	69/70 (98.6)	60/64 (93.8)	56/69 (81.2)	<.001
PCR- or serology-confirmed *R. typhi*	136/146 (93.2)	53/53 (100)	45/47 (95.7)	38/46 (82.6)	<.001
PCR-confirmed *R. typhi*	47/49 (95.9)	17/17 (100)	18/18 (100)	12/14 (85.7)	.08
No PCR or confirmed serologic evidence of R. typhi	49/57 (86.0)	16/17 (94.1)	15/17 (88.2)	18/23 (78.3)	.41
FCT, median (IQR), h^c^					
All patients	37 (24–66)	34 (24–58)	36 (24–51)	48 (24–96)	.002
PCR- or serology-confirmed *R. typhi*	36 (24–60)	32 (24–48)	36 (24–60)	43 (20–107)	.02
PCR-confirmed *R. typhi*	48 (30–66)	42 (30–60)	34 (24–60)	66 (48–162)	.005
No PCR or confirmed serologic evidence of R. typhi	42 (24–78)	36 (24–68)	30 (24–50)	70 (24–100)	.07
AUC,^d^ median (IQR), °C * h					
All	1368 (891–2259)	1243 (891–2016)	1312 (792–1923)	1639 (892–2648)	.056
PCR- or serology-confirmed *R. typhi*	1370 (895–2249)	1211 (903–1834)	1356 (681–2233)	1616 (743–2448)	.26
PCR-confirmed *R. typhi*	1827 (1130–2465)	1591 (1126–2247)	1312 (920–2267)	2360 (1827–4719)	.02
No PCR or confirmed serologic evidence of R. typhi	1363 (891–2499)	1346 (455–2186)	1117 (911–1634)	1845 (892–3045)	.20

Abbreviations: AUC, area under the time-temperature curve; FCT, fever clearance time; IQR, interquartile range; PCR, polymerase chain reaction.

^a^Frequencies were compared using χ^2^ tests across 3 groups (ie, 2 degrees of freedom), clearance times were compared using log-rank tests, and AUC estimates were compared using Kruskal-Wallis tests.

^b^Treatment failure was assessed in 213 patients; 3 patients withdrew, 1 in each arm, before treatment failure/success could be defined.

^c^Of 203 patients who presented with or developed fever, fever did not clear before discharge (or withdrawal) in 18; excluded are 12 patients who were afebrile at admission and 1 who withdrew at hour 0.

^d^AUC for period up to FCT or last patient follow up, if fever not cleared (excluding 12 patients with no fever at admission and 1 who withdrew at hour 0). AUCs for 4 patients afebrile at admission who developed fever during follow-up were calculated from the time of first temperature ≥37.5°C.

The time-temperature AUC and FCT were also significantly larger and longer, respectively, for patients in the A3 group with PCR-confirmed *R. typhi* (*P* = .02). Kaplan-Meier analysis demonstrated longer FCT for the A3 than for the D7 and D3 groups (Figures 3–5; log-rank *P* < .02 for all diagnostic groups). There were no significant differences between D3 and D7 groups for any of the outcomes (all *P* ≥ .20) and no significance difference in the risk of failure between younger (≤40 years) and older (>40 years) patients (*P* = .11). Of the 103 patients with adverse event data at Mahosot Hospital, 59 (57%) had mild adverse events, excluding headache, which was a consistent feature of murine typhus (rash occurred in 15 of 93 [16.1%], diarrhea in 14 of 84 [16.7%], vomiting in 12 of 89 [13.5%]), with no significant difference between the 3 treatment groups (*P* = .06).

The median (IQR, range) duration of follow-up was 208 days (IQR, 165–369 days; range, 2–666 days) with 99 (55%) of the Mahosot Hospital patients completing 1 year. Twenty-six patients (12.0%) returned with fever (Supplementary Material); none were thought clinically to have murine typhus relapse. Of 14 patients with repeated *R. typhi* PCR performed on readmission buffy coats all were negative. All returning patients recovered without antirickettsial antibiotic therapy and were well at discharge.

## DISCUSSION

The results of this trial suggest that azithromycin is inferior to doxycycline as oral therapy for uncomplicated murine typhus. The clinical outcome measures of FCT, AUC, and frequency of treatment failure were significantly and consistently inferior for the azithromycin group compared with the doxycycline groups. Although the D3 group had longer FCTs, higher treatment failure frequency, and larger AUC than the D7 group, **t**here were no significant differences (*P* ≥ .05) (Table 4), suggesting that 3 days of doxycycline is adequate for treating uncomplicated murine typhus in Lao adults. Owing to our limited understanding of the geographic genomic, antimicrobial resistance variability of and human susceptibility to *R. typhi,* this conclusion can be generalized only with caution. These data have implications for the use of azithromycin in the empirical treatment of fever in communities where murine typhus is common [22].

There have been concerns that murine typhus may relapse, especially after early brief chloramphenicol therapy [23, 24], perhaps because of treatment discontinuation before development of an effective immune response. In Laos we found no evidence of relapse, but active, rather than passive, long-term patient follow-up is required. Epidemic typhus is known to relapse, as Brill-Zinsser disease, but the interval between an initial attack of epidemic typhus and relapse is measured in years and not months [25].

Limitations of the current trial include the fact that we did not assay the quality of the study antibiotics before or during the trial, but they were manufactured by a major innovative pharmaceutical company and stored appropriately. We did not follow up patients actively after discharge. Even though murine typhus is a global disease, diagnostics have been neglected and are inadequate. There is no consensus on appropriate IFA murine typhus diagnostic cutoff titers for different levels of endemicity. Patients usually present with low *R. typhi* bacterial blood loads, and there has been minimal research on improvements in diagnostic assays [26].

We chose an azithromycin dose of 500 mg followed by 250 mg once a day for 2 days, based on evidence available for scrub typhus [27, 28]. A randomized trial in Korean patients with scrub typhus found that a single 500-mg azithromycin dose was as efficacious, in terms of fever clearance and relapse, as doxycycline at 200 mg/d for 7 days [28]. To our knowledge, no trials have compared different azithromycin dose regimens for any rickettsial disease.

These data have particular bearing for pregnant women with murine typhus [14]. It has been argued that doxycycline could be considered less stringently as therapy in pregnancy and children, because no correlation was found between the use of doxycycline and teratogenic effects during pregnancy or dental staining in children [29]. However, recent evidence has associated both doxycycline and azithromycin with spontaneous abortions [30].

Interestingly, 2 patients with Brill-Zinsser disease have been reported with failure of oral azithromycin therapy (500 mg/d for 3 days) [31]. Because *R. typhi* and *R. prowazekii* are closely related, these failures may have a similar mechanism to those we report. Acquired azithromycin resistance in other human pathogens, such as *Mycoplasma pneumonia* and *Neisseria gonorrhoeae,* is mediated by the ribosomal target of the antibiotic, 23S ribosomal RNA [32]. We are aware of no equivalent data for *R. typhi,* and investigations of genomic (especially 23S ribosomal RNA) and phenotypic markers of resistance to azithromycin, and 14-membered lactone ring macrolides, are urgently needed.

## Supplementary Data

Supplementary materials are available at *Clinical Infectious Diseases* online. Consisting of data provided by the authors to benefit the reader, the posted materials are not copyedited and are the sole responsibility of the authors, so questions or comments should be addressed to the corresponding author.

Supplementary Material

## Notes

***Acknowledgments.*** We are very grateful to all the patients who participated in this study and the physicians, nursing staff, and technicians of the infectious diseases ward and the microbiology laboratory, especially Simmaly Phongmany, Anisone Changthongthip, Sengmany Symanivong, and Matthew Robinson. We thank Ampai Tanganuchitcharnchai and Suthatip Jintaworn for performing IFA assays and Liz Ashley and Tri Wangrangsimakul for helpful comments. Ric Price kindly provided invaluable help as trial monitor and advisor. We are very grateful to the Minister of Health, H.E. Professor Bounkong Syhavong, and Dr Bounnak Xaysanasongkham, Director General of the Department of Health Care and Rehabilitation, Ministry of Health, for their support for this study, which was part of the Lao-Oxford-Mahosot Hospital–Wellcome Trust Research Unit.

***Financial support.*** This work was supported by the Wellcome Trust.

***Potential conflicts of interest.*** All authors: No reported conflicts of interest. All authors have submitted the ICMJE Form for Disclosure of Potential Conflicts of Interest. Conflicts that the editors consider relevant to the content of the manuscript have been disclosed.
