# Supplementary material for: A Prospective, Open-label, Randomized Trial of Doxycycline Versus Azithromycin for the Treatment of Uncomplicated Murine Typhus
Source: Clin Infect Dis. 2018 Jul 18;68(5):738–47. doi: 10.1093/cid/ciy563 (PMC6376095; doi:10.1093/cid/ciy563)
Supplement: Supplementary Material [file ciy563_suppl_supplementary_material.docx]

A prospective, open-label, randomised trial in Lao adults with uncomplicated murine typhus: seven versus three days doxycycline versus three days azithromycin

**SUPPLEMENTARY MATERIAL**

Supplementary Material Text S1

IgM and IgG antibodies against *Rickettsia typhi*were detected using indirect immunofluorescence assays (IFA) after the study was completed. Testing was performed using slides coated with *Rickettsia typhi*Wilmington antigen (Australian Rickettsial Reference Laboratory, Geelong, Australia). Patient sera were serially 2-fold diluted from 1:400 to 1:3200 and the endpoint was determined as the highest titre displaying specific fluorescence. Positivity was defined as a 1) 4-fold or greater rising titer in IgM or IgG antibodies when comparing admission to subsequent longitudinal samples, and/or 2) a positive reciprocal titer of >3200 in an admission sample.

Genomic DNA extraction was performed using QIAamp DNA Mini Kit (Qiagen, Germany) according to the manufacturer's instructions except the incubation time at 56°C was increased to 1 hour. The resulting gDNA was kept in supplied elution buffer and stored at -20°C until use. PCR reactions were carried out as follows. Briefly, 1 µl of gDNA extract was added to a total volume of 25 μL of Platinum Quantitative PCR SuperMix-UDG (Thermo Fisher Scientific, USA) containing primers specific to the 47kDa gene of *O. tsutsugamushi* [[1](#_ENREF_1)] or 17kDa gene of *Rickettsia* spp. [[1](#_ENREF_1)]. The thermal profile used for amplification was 2 min at 50°C, 2 min at 95°C, followed by 45 cycles of 15 sec at 95°C and 30 sec at 60°C. Bacterial loads were determined using the standard curves of known copy numbers obtained with the 47kDa-based or *ompB*-based linearized plasmids as described [[2](#_ENREF_2)]. Upon successful amplification of the 17kDa gene, *Rickettsia* spp. identification was performed using the *R. typhi-*specific PCR targeting the *ompB* gene [[3](#_ENREF_3)] or by nested PCR and sequencing based on the 17kDa gene [[4](#_ENREF_4)]. Platinum Taq DNA Polymerase (Thermo Fisher Scientific, USA) system was used in a 50 μL reaction containing dNTPs, MgCl_2_, Platinum Taq buffer, and 17kDa-specific primers. The conditions used for first round amplification were 1 min at 94°C and 35 cycles of 30 sec at 94°C, 30 sec at 55°C, and 2 min at 68°C with a final 7 min at 72°C to allow complete extension of the PCR product. The second round reactions were carried out similarly except the annealing temperature was increased to 58°C and extension time decreased to 1.5 min. PCR products were visualized on 1% ethidium bromide-stained TBE agarose gel with the expected size of 434 bp. Species identification was accomplished through a BLAST search against known sequences of Rickettsia species. For spotted fever group, 1 μL of DNA template was added to a total volume of 25 μL PCR mix containing Platinum Taq DNA polymerase, dNTP, MgCl_2_, Platinum Taq Buffer, and primers specific to the *ompB* gene [[5](#_ENREF_5)]. The conditions used for amplification were 1 min at 94°C followed by 35 cycles of 30 sec at 94°C, 30 sec at 54°C, and 30 sec at 68°C with an additional 7 min at 72°C at the end of the run. PCR products were visualized on 1% Ethidium bromide-stained TBE agarose gel with the expected size of 440 bp. Species identification was performed via BLAST analysis, as above. For *Leptospira* spp. detection, 5 uL of gDNA extract was added to a total volume of 20 μL PCR mix containing Platinum Quantitative PCR SuperMix-UDG (Thermo Fisher Scientific, USA), MgCl_2_, and primers specific to the *rrs* gene [[6](#_ENREF_6)]. The conditions used for amplification were 2 min at 50°C, 8 min at 95°C, and 45 cycles of 15 sec at 95°C and 1 min at 60°C.

Supplementary Material S1 - References

1. Jiang J, Chan TC, Temenak JJ, Dasch GA, Ching WM, Richards AL: Development of a quantitative real-time polymerase chain reaction assay specific for *Orientia tsutsugamushi***.** *Am J Trop Med Hyg* 2004, **70:**351-356.

2. Dittrich S, Castonguay-Vanier J, Moore CE, Thongyoo N, Newton PN, Paris DH: Loop-mediated isothermal amplification for *Rickettsia typhi* (the causal agent of murine typhus): problems with diagnosis at the limit of detection**.** *J Clin Microbiol* 2014, **52:**832-838.

3. Henry KM, Jiang J, Rozmajzl PJ, Azad AF, Macaluso KR, Richards AL: Development of quantitative real-time PCR assays to detect *Rickettsia typhi* and *Rickettsia felis*, the causative agents of murine typhus and flea-borne spotted fever. *Mol Cell Probes* 2007, **21:**17-23.

4. Taylor AJ, Vongphayloth K, Vongsouvath M, Grandadam M, Brey PT, Newton PN, Sutherland IW, Dittrich S: Large-Scale Survey for Tickborne Bacteria, Khammouan Province, Laos**.** *Emerg Infect Dis* 2016, **22:**1635-1639.

5. Jiang J, Blair PJ, Felices V, Moron C, Cespedes M, Anaya E, Schoeler GB, Sumner JW, Olson JG, Richards AL: Phylogenetic analysis of a novel molecular isolate of spotted fever group Rickettsiae from northern Peru: Candidatus *Rickettsia andeanae*. *Ann N Y Acad Sci* 2005, **1063:**337-342.

6. Thaipadungpanit J, Chierakul W, Wuthiekanun V, Limmathurotsakul D, Amornchai P, Boonslip S, Smythe LD, Limpaiboon R, Hoffmaster AR, Day NP, Peacock SJ: Diagnostic accuracy of real-time PCR assays targeting 16S rRNA and lipL32 genes for human leptospirosis in Thailand: a case-control study. *PLoS One* 2011, **6:**e16236.

**Table S1. Details of patients classed as treatment failures**

| **Patient Code** | **When classed as treatment failure/h** | **Clinical details at time of treatment failure** | **Treatment & outcome** | **Diagnosis** | **Notes** |
| --- | --- | --- | --- | --- | --- |
| **Doxycycline 7 days** | | | | |  |
| MUT 22 | 70 | 37.8^o^C & unwell | Changed to 3 days oral ofloxacin at 70 h when found to be *S.* Typhi blood culture positive. Doxycycline continued for 7 days. Discharged well | Blood cultures grew *S.* Typhi |  |
| **Doxycycline 3 days** | | | | |  |
| MUT 15 | 84 | 38.5^o^C & unwell | Continued doxycycline to complete 7 day course. Discharged well | PCR positive *R. typhi* |  |
| MUT 24 | 72 | 37.6^o^C & unwell | Continued doxycycline to complete 7 day course. Discharged well | Serological diagnosis of *R. typhi* |  |
| MUT 62 | 90 | 37.9^o^C & unwell | Continued doxycycline to complete 7 day course. Discharged well | No diagnosis  made |  |
| **Azithromycin 3 days** | | | | |  |
| MUT 4 | 74 | 37.9^o^C & unwell with headache, myalgia | 7 days doxycycline started at 78 h. Discharged well | PCR positive *R. typhi* |  |
| MUT 5 | 72 | 39.0^o^C & unwell, headache not improved | 7 days doxycycline started at 78 h. Discharged well | No diagnosis  made |  |
| MUT 13 | 90 | 38.0^o^C & unwell, headache, myalgia | 7 days doxycycline started at 92 h. Discharged well | PCR positive *R. typhi*. HbH & G6PD deficient |  |
| MUT 28 | 72 | 38.9^o^C | 7 days doxycycline started at 79h. Discharged well | PCR positive *R. typhi* |  |
| MUT 37 | 49 | 38^o^C | 4FDC started at 49 h with report that sputum acid-fast bacilli positive. Recovered | Pulmonary TB |  |
| MUT 67 | 134 | 37.6^o^C with headache | 7 days doxycycline started at 139 h. Discharged well | Serological diagnosis of *R. typhi* |  |
| MUT 92 | 90 | 38.6 ^o^C, unwell, with headache, malaise & myalgia | 7 days doxycycline started at 90h. Discharged well | Serological diagnosis of *R. typhi* |  |
| MUT 96 | 144 | 39.3 ^0^C, unwell, headache | 7 days doxycycline started at 144 h. Discharged well | No diagnosis  made |  |
| MUT 129 | 90 | 38.0 ^o^C, unwell | 7 days doxycycline started at 90 h. Discharged well | Serological diagnosis of *R. typhi* |  |
| MUT 144 | 78 | 38.1 ^o^C, unwell with headache | 7 days doxycycline started at 78 h.  Discharged well | PCR positive *R. typhi* |  |
| MUT 146 | 114 | 38.0 ^0^C, headache, myalgia | 7 days doxycycline started at 120 h | No diagnosis  made |  |
| MUT 156 | 36 | 37.8 ^o^C, unwell | Given doxycycline and ceftriaxone in Thai hospital. Discharged well | Probable serological diagnosis of *R. typhi* | Self-discharged to Thai hospital |
| MUT 165 | 72 | 38.4 ^0^C, unwell | 7 days doxycycline started at 72 h | No diagnosis  made | Patient self-discharged and took total of 4 days doxycycline. Recovered |
| MUT 171 | 90 | 38.4 ^0^C, headache, myalgia, fever, cough with lung crepitations | 7 days doxycycline started at 92 h. Discharged well | PCR positive *R. typhi* |  |
| MUT 205 | 108 | 37.6 ^0^C, headache myalgia, unwell | 7 days doxycycline started at 114 h. Discharged well | PCR positive *O. tsutsugamushi* |  |
| MUT 208 | 90 | 38.6 ^0^C and headache | 7 days doxycycline started at 96 h. Discharged well | PCR positive *R. typhi* |  |

**Table S2. Details of protocol violations and patient withdrawals**

| **Patient Code** | **Details** | **Diagnosis** |
| --- | --- | --- |
|  | | |
| **Doxycycline 7 days** | | |
| MUT 2 | Discharged with last two doxycycline 100mg capsules not taken | Serological diagnosis of *R. typhi* |
| MUT 58 | Discharged with last 2 doxycycline capsules not taken as diagnosed with pulmonary TB | Pulmonary TB diagnosed by X-Ray |
| MUT 71 | Discharged with last doxycycline 100mg capsule not taken | Serological diagnosis of *R. typhi* |
| MUT 85 | Given antacid once a day on first and second days of doxycycline | PCR positive *R. typhi* |
| MUT 139 | Vomited 5 mins after loading dose but not redosed. Given one dose of antacid on third day of doxycycline | PCR positive *R. typhi* |
| MUT 181 | Went home 2 days before course completion, after fever clearance time, with remaining full course – but unknown if took them. Three doses of antacid given on second and fourth day of doxycycline | Serological diagnosis of *R. typhi* |
| MUT 186 | Patient received doxycycline 200mg loading dose but one hour later family asked to take patient to Thailand. At Nong Khai Hospital febrile for first 2 days- doctors only gave antipyretics and told patient had viral infection. Classed as patient withdrawal. | PCR positive *R. typhi* |
| MUT 191 | Given antacid every 12 hours on third and fourth day of doxycycline course | Serological diagnosis of *R. typhi* |
| **Doxycycline 3 days** | | |
| MUT 1 | No loading dose of doxycycline given - 100mg and not 200mg as first dose | PCR positive *R. typhi* |
| MUT 6 | Received one non-study doxycycline 100mg capsule during course | PCR positive *R. typhi* |
| MUT 41 | Patient self-transferred to Thailand, before fever clearance at 24h. Had additional 16 doxycycline 100mg capsules in Thailand of non-study drug and patient recovered. Classed as patient withdrawal. | PCR positive *O. tsutsugamushi* |
| MUT 44 | Given antacid three times a day on second, third and fourth days of doxycycline | Serological diagnosis of *R. typhi* |
| MUT 50 | Given one dose of antacid on third day of doxycycline | No diagnosis  made |
| MUT 76 | Discharged with last doxycycline 100mg capsule not taken | Serological diagnosis of *R. typhi* |
| MUT 97 | One additional 100mg doxycycline capsule given | PCR positive *R. typhi* |
| MUT 104 | One additional 100mg doxycycline capsule given | Serological diagnosis of *R. typhi* |
| MUT 126 | Given antacid on first and second days of doxycycline | PCR positive *R. typhi* |
| MUT 147 | One additional 100mg doxycycline capsule given | Serological diagnosis of *R. typhi* |
| MUT 187 | Given antacid on first, third and fourth days of doxycycline | Serological diagnosis of *R. typhi* |
| **Azithromycin 3 days** | | |
| MUT 13 | Not given one doxycycline 100mg capsule during retreatment after failing azithromycin 3 days therapy | PCR positive *R. typhi* |
| MUT 196 | Patient improving but still febrile at 48h with bad headache and his mother asked him to go to Thailand. No information from Thai hospital. Too early for FCT treatment failure judgement at 72 h. Classed as patient withdrawal. | No diagnosis  made |

All the antacids given were Amacone = aluminium hydroxide, magnesium hydroxide, semethicone suspension

**Table S3. Details of patients readmitted unwell during one year follow up**

| **Patient Code/**  **Antibiotic therapy/**  **Admission diagnosis** | **Interval between admission and readmission /days**  **Month of readmission** | **Clinical details at readmission** | **Diagnosis on readmission/*R. typhi* & *O. tsutsugamushi* PCR result/**  **Diagnosis at discharge** | **Treatment & outcome** |
| --- | --- | --- | --- | --- |
| MUT 9  Doxycycline 7 days  *R. typhi* PCR positive | 320  March | 2 days fever, headache, arthralgia, myalgia. Chest, abdomen & heart examination normal. Pharyngitis with pus on left tonsil. Admitted. Fever while in hospital | Very unlikely to be relapse  N/A  Tonsillitis | Penicillin V & paracetamol. Discharged well |
| MUT 11  Azithromycin 3 days  Serological diagnosis of *R. typhi* | 272  February | 2 days fever, headache, arthralgia, myalgia, diarrhoea, dyspnoea, sore throat. Admitted - highest temperature = 37.4 ^o^C | Unlikely to be relapse  N/A  Influenza | No antibiotics. Discharged well |
| MUT 14  Doxycycline 7 days  Serological diagnosis of *R. typhi* | 116  September | 3 days fever, myalgia, arthralgia, headache, nausea, sore throat and rhinitis, dry cough | Very unlikely to be relapse  Negative  Influenza | No antibiotics. Discharged well |
| MUT 20  Doxycycline 3 days.  Probable serological diagnosis of *O. tsutsugamushi* | 14  June | Returned with fever. Not admitted | Undetermined  N/A | Paracetamol and improved rapidly |
| MUT 22  Doxycycline 7 days  *S*. Typhi blood culture positive | 361  May | 4 days headache, fever, abdominal pain, without hepatosplenomegaly. Admission 39.0^0^C, enlarged tonsils but no pus, WCC 7.3 10^9^/L, malaria smear negative | Unlikely to be relapse  Negative  Influenza | Paracetamol. No antibiotics  Discharged well |
| MUT 25  Doxycycline 3 days  No diagnosis | 56  July | 3 days fever, 38.5 ^o^C. WCC 13.5 10^9^/L. | Unlikely to be relapse  N/A  Influenza | Paracetamol. No antibiotics  Discharged well |
| MUT 31  Azithromycin  3 days  *O. tsutsugamushi* PCR positive | 8  July | 4 days fever, headache, chill. CXR normal. | Unlikely to be relapse  Negative  No diagnosis | Paracetamol. No antibiotics  Discharged well |
| MUT 31  Second readmission | 120  November | Cough, headache, epixtaxis, haematemesis x 1. No fever during admission. | Not relapse  N/A  No diagnosis | Paracetamol. No antibiotics  Discharged well |
| MUT 33  Doxycycline 3 days  Serological diagnosis of *O. tsutsugamushi* | 144  December | 2 days fever and tiredness. No fever whilst inpatient for 2 days  Patient had been admitted to parallel scrub typhus clinical trial 8th October 2003 | Very unlikely to be relapse  N/A  ‘Viral illness’ | No treatment  Discharged well |
| MUT 38  Doxycycline 3 days  Serological diagnosis of *R. typhi* | 187  January | 2 days fever & headache. Admitted for one day but patient self-discharged. No treatment or investigations. | Very unlikely to be relapse  Negative  No diagnosis | No treatment  Telephoned 3 days after self discharge and said that he had no fever but had headache |
| MUT 45  Azithromycin  3 days  Probable serological diagnosis of *R. typhi* | 196  March | 3 days fever, headache, myalgia, arthralgia, cough & sputum. T = 38 ^o^C, crepitations at left lung base. WCC 5.0 10^9^/L, malaria smear negative | Unlikely to be relapse  N/A  ‘Viral infection’ | Paracetamol, dexchlorpheniramine & vitamin C. No antibiotics  Discharged well |
| MUT 48  Azithromycin 3 days  No diagnosis | 84  December | 1 day fever, sore throat, headache, enlarged red tonsils with normal peripheral blood cell count. Not admitted | Unlikely to be relapse  N/A  Probable viral pharyngitis | No treatment  Discharged well |
| MUT 54  Doxycycline 7 days  *O. tsutsugamushi* PCR positive | 162  April | 4 days fever, headache, nausea, myalgia, vomiting, 38.0 ^0^C, white count 13.0 10^9^/L | Possible relapse  Negative  Influenza? | Paracetamol. No antibiotics  Discharged well |
| MUT 55  Azithromycin 3 days  Serological diagnosis of *R. typhi* | 25  November | 7 days chest pain when eats, tired and with headache, paraesthesia of feet and hands. No fever. Admitted one night | Very unlikely to be relapse  N/A  No diagnosis | Discharged well |
| MUT 62  Doxycycline 3 days  No diagnosis | 231  August | 3 days of fever, headache, myalgia and diarrhoea, 38.1 ^0^C, conjunctival suffusion. Admitted, fever resolved in 48 hours | Very unlikely to be relapse  Negative  Influenza | Paracetamol. No antibiotics  Discharged well |
| MUT 72  Azithromycin 3 days  Serological diagnosis of *R. typhi* | 238  July | 3 days headache, myalgia, cough and yellow sputum, fever, bilateral parotid swelling, respiratory wheeze, white count 9.6 10^9^/L, malaria smear negative | Very unlikely to be relapse  Negative  Influenza | Paracetamol & dexchlorpheniramine. No antibiotics.  Discharged well |
| MUT 73  Doxycycline 7 days  No diagnosis | 17  December | 3 days rhinorrhoea, cough, white sputum, without fever & chest clear. | Very unlikely to be relapse  N/A  Upper respiratory tract infection | No treatment  Discharged well |
| MUT 78  Azithromycin 3 days  *R. typhi* PCR positive | 190  September | 5 days headache, myalgia & fever, retro-orbital pain & constipation, 39.7 ^o^C, chest clear, pharynx normal, white count 8.6 10^9^/L, malaria film negative | Unlikely to be relapse  Negative  Influenza/viral infection | Paracetamol & dexchlorpheniramine. No antibiotics.  Discharged well |
| MUT 80  Doxycycline 3 days  Serological diagnosis of *R. typhi* | 279  January | 3 days fever, headache, myalgia, cough, rhinitis, sore throat, diarrhoea, nausea & vomiting, 37.2 ^o^C, white count 7.4 10^9^/L. No fever whilst inpatient | Unlikely to be relapse  N/A  Influenza | Paracetamol. No antibiotics.  Discharged well |
| MUT 89  Doxycycline 7 days  Serological diagnosis of *R. typhi* | 360  June | 2 days fever, headache, myalgia, sore throat, swollen tonsils with white patches, 39.4 ^o^C, white count 9.8 10^9^/L, malaria smear negative. Fever resolved in 36 h | Very unlikely to be relapse  N/A  Tonsillitis | Paracetamol & oral amoxicillin  Discharged well |
| MUT 93  Doxycycline 3 days  *R. typhi* PCR positive | 19  August | 10 days headache, myalgia, rhinorrhoea, dysphagia, cough with yellow/green sputum, 37.7 ^o^C, red pharynx. No treatment, Fever < 37.5 after 12 h. | Unlikely to be relapse  Negative  Upper respiratory tract infection | No treatment  Discharged well |
| MUT 96  Azithromycin 3 days  No diagnosis | 59  January | 3 days fever, sore throat, nasal congestion, white count 6,000 10^9^/L, malaria smear negative. No fever recorded in 2 days in hospital | Unlikely to be relapse  Negative  Influenza | Paracetamol & chlorphenamine. No antibiotics  Discharged well |
| MUT 129  Azithromycin 3 days  Serological diagnosis of *R. typhi* | 54  September | 1 day fever, headache, dyspnoea, dysphagia, cough with sputum, myalgia, 38.4 ^o^C, white count 12.6 10^9^/L, malaria smear negative. | Unlikely to be relapse  Negative  Influenza | Paracetamol, dexchlorpheniramine. No antibiotics  Discharged well |
| MUT 138  Doxycycline 3 days  Serological diagnosis of *R. typhi* | 250  September | 1 day fever, cough, runny nose, myalgia, red face, headache, 37.5 oC, red throat. White count 10.7 10^9^/L, malaria negative | Very unlikely to be relapse  N/A  Viral infection | Paracetamol, vitamin C. No antibiotics  Discharged well |
| MUT 160  Azithromycin 3 days  Serological diagnosis of *R. typhi* | 234  January | 2 days fever, headache, runny nose. Physical exam normal. | Very unlikely to be relapse  Negative |  |
| MUT 193  Doxycycline 7 days  *R. typhi* PCR positive | 21  February | 3 days fever, sore throat, cough with green sputum, myalgia, dyspnoea, weakness, lungs examination normal. White count =5.9 10^9^/L | Unlikely to be relapse  Negative  Influenza | Paracetamol. No antibiotics  Discharged well |
| MUT 200  Doxycycline 3 days  No diagnosis | 11  April | Dizzyness, tired, tinnitus but no fever. No fever during 4 days admission. | Very unlikely to be relapse  Negative  Vertigo | Vitamin B complex. No antibiotics  Discharged well |

White count = peripheral venous blood white cell count

N/A = not available

**Consort Checklist**

CONSORT 2010 checklist of information to include when reporting a randomised trial*

| Section/Topic | Item No | Checklist item | Reported on page No |
| --- | --- | --- | --- |
| Title and abstract | | | |
|  | 1a | Identification as a randomised trial in the title | 1 |
|  | 1b | Structured summary of trial design, methods, results, and conclusions (for specific guidance see CONSORT for abstracts) | 2 |
| Introduction | | | |
| Background and objectives | 2a | Scientific background and explanation of rationale | 4-5 |
|  | 2b | Specific objectives or hypotheses | 4-5 |
| Methods | | | |
| Trial design | 3a | Description of trial design (such as parallel, factorial) including allocation ratio | 5 |
|  | 3b | Important changes to methods after trial commencement (such as eligibility criteria), with reasons | N/A |
| Participants | 4a | Eligibility criteria for participants | 5-6 |
|  | 4b | Settings and locations where the data were collected | 5 |
| Interventions | 5 | The interventions for each group with sufficient details to allow replication, including how and when they were actually administered | 7 |
| Outcomes | 6a | Completely defined pre-specified primary and secondary outcome measures, including how and when they were assessed | 7-8 |
|  | 6b | Any changes to trial outcomes after the trial commenced, with reasons | N/A |
| Sample size | 7a | How sample size was determined | 8 |
|  | 7b | When applicable, explanation of any interim analyses and stopping guidelines | 8 |
| Randomisation: |  |  |  |
| Sequence generation | 8a | Method used to generate the random allocation sequence | 7 |
|  | 8b | Type of randomisation; details of any restriction (such as blocking and block size) | 7 |
| Allocation concealment mechanism | 9 | Mechanism used to implement the random allocation sequence (such as sequentially numbered containers), describing any steps taken to conceal the sequence until interventions were assigned | 7 |
| Implementation | 10 | Who generated the random allocation sequence, who enrolled participants, and who assigned participants to interventions | 7 |
| Blinding | 11a | If done, who was blinded after assignment to interventions (for example, participants, care providers, those assessing outcomes) and how | N/A |
|  | 11b | If relevant, description of the similarity of interventions |  |
| Statistical methods | 12a | Statistical methods used to compare groups for primary and secondary outcomes | 8 |
|  | 12b | Methods for additional analyses, such as subgroup analyses and adjusted analyses |  |
| Results | | | |
| Participant flow (a diagram is strongly recommended) | 13a | For each group, the numbers of participants who were randomly assigned, received intended treatment, and were analysed for the primary outcome | 9/10, Fig 1 |
|  | 13b | For each group, losses and exclusions after randomisation, together with reasons | Fig 1 |
| Recruitment | 14a | Dates defining the periods of recruitment and follow-up | 9 |
|  | 14b | Why the trial ended or was stopped | 9 |
| Baseline data | 15 | A table showing baseline demographic and clinical characteristics for each group | Table 3 |
| Numbers analysed | 16 | For each group, number of participants (denominator) included in each analysis and whether the analysis was by original assigned groups | Table 3 |
| Outcomes and estimation | 17a | For each primary and secondary outcome, results for each group, and the estimated effect size and its precision (such as 95% confidence interval) | Table 4 |
|  | 17b | For binary outcomes, presentation of both absolute and relative effect sizes is recommended | N/A |
| Ancillary analyses | 18 | Results of any other analyses performed, including subgroup analyses and adjusted analyses, distinguishing pre-specified from exploratory | N/A |
| Harms | 19 | All important harms or unintended effects in each group (for specific guidance see CONSORT for harms) | N/A |
| Discussion | | | |
| Limitations | 20 | Trial limitations, addressing sources of potential bias, imprecision, and, if relevant, multiplicity of analyses | 12/13 |
| Generalisability | 21 | Generalisability (external validity, applicability) of the trial findings | 12 |
| Interpretation | 22 | Interpretation consistent with results, balancing benefits and harms, and considering other relevant evidence | 12/13 |
| Other information | | |  |
| Registration | 23 | Registration number and name of trial registry | ISRCTN 47812566 |
| Protocol | 24 | Where the full trial protocol can be accessed, if available | N/A |
| Funding | 25 | Sources of funding and other support (such as supply of drugs), role of funders | 2 |
